# PDLIM3 Regulates Migration and Invasion of Head and Neck Squamous Cell Carcinoma via YAP–Mediated Epithelial–Mesenchymal Transition

**DOI:** 10.3390/ijms26073147

**Published:** 2025-03-28

**Authors:** Fan Yang, Ying Zhou, You Zhang, Weideng Wei, Fei Huang, Dan Yang, Yixin Zhang, Ruiyang Zhang, Xiaoqiang Xia, Qianming Chen, Yuchen Jiang, Xiaodong Feng

**Affiliations:** State Key Laboratory of Oral Diseases & National Center for Stomatology & National Clinical Research Center for Oral Diseases & Frontier Innovation Center for Dental Medicine Plus & Research Unit of Oral Carcinogenesis and Management & Chinese Academy of Medical Sciences, West China Hospital of Stomatology, Sichuan University, Chengdu 610041, China; fanyang2022@stu.scu.edu.cn (F.Y.); 2020324030006@alu.scu.edu.cn (Y.Z.); u20230320@outlook.com (Y.Z.); weiweideng@stu.scu.edu.cn (W.W.); antigenjerry@163.com (F.H.); ydan1@stu.scu.edu.cn (D.Y.); 2020224035084@stu.scu.edu.cn (Y.Z.); ruiyang0910@163.com (R.Z.); xxq_312@163.com (X.X.); qmchen@scu.edu.cn (Q.C.)

**Keywords:** HNSCC, PDLIM3, prognostic biomarker, tumor metastatic capability, EMT, cytoskeleton remodeling, YAP

## Abstract

Despite significant progress in characterizing the omics landscape of head and neck squamous cell carcinoma (HNSCC), the development of precision therapies remains limited. One key factor contributing to this challenge is the marked molecular heterogeneity of HNSCC. Further investigation of molecular profiles within HNSCC may facilitate the improvement in more effective precision treatments. Here, we focus on the dysregulation of PDZ and LIM domain protein 3 (PDLIM3) in HNSCC. The expression levels of PDLIM3 were analyzed using public datasets to assess its potential role in tumor progression. We found that PDLIM3 was downregulated in pan–cancer and HNSCC. The prognostic significance of PDLIM3 was evaluated through tissue microarray, and the downregulation of PDLIM3 was correlated with poor HNSCC prognosis. Investigating the implications of PDLIM3 for tumor metastatic ability in vitro, we found that PDLIM3 suppressed the migration and invasion of HNSCC, accompanied by partially impeding the process of epithelial–mesenchymal transition (EMT). Furthermore, PDLIM3 inhibited the transcriptional activity of Yes–associated protein (YAP), suggesting that YAP may be involved in the PDLIM3–mediated suppression of HNSCC metastatic ability. Our findings identify a potential signaling axis wherein PDLIM3 regulates YAP–EMT, thereby influencing tumor metastatic ability, and suggest the potential role of PDLIM3 as a tumor suppressor and prognostic biomarker for HNSCC.

## 1. Introduction

Head and neck squamous cell carcinoma (HNSCC) is the sixth most prevalent cancer worldwide [1,2] and is the most common malignant tumor among head and neck cancers [3,4]. Broad–spectrum therapies remain the first–line treatment option. However, their efficacy is limited [5]. Approximately 50–60% of patients with locally advanced HNSCC experience recurrence or metastasis [3,6], which significantly contributes to poor prognosis and postoperative treatment failure [7,8]. Additionally, the marked molecular heterogeneity makes the development of targeted therapies difficult [3,9]. Despite significant progress in characterizing the omics landscape of HNSCC, our understanding of the highly heterogeneous HNSCC remains inadequate, and the development of precision therapies remains limited [4,6]. These unfavorable outcomes have driven further research into the aberrant molecular events and related mechanisms of HNSCC [10,11], with the goal of identifying and providing novel prognostic biomarkers and therapeutic targets to improve therapeutic outcomes and patient prognosis.

The dysregulation of PDZ and LIM domain protein 3 (PDLIM3) in pan–cancer indicates its significance in tumor progression [12]. PDLIM3 is a scaffold protein that contains a PDZ domain and a LIM domain, classifying it within the PDZ–LIM family proteins [12,13,14]. Previous studies have emphasized its important roles in cytoskeletal remodeling [15], muscle development [15,16], and signal transduction [17]. However, recent research has revealed aberrant expression of PDLIM3 in various cancers. PDLIM3 is highly expressed in the sonic hedgehog (SHH) group of medulloblastoma (MB) [18] and in gastric cancer [19], while its expression is significantly reduced in prostate cancer [20]. The inconsistent expression patterns of PDLIM3 highlight the complexity of its regulatory mechanisms in cancers. However, its role in the progression of HNSCC remains poorly understood.

Above all, we hypothesized that PDLIM3 plays a crucial role in the progression of HNSCC. Our study aims to evaluate its biological contribution and clinical value in tumor progression by analyzing the expression levels of PDLIM3 in HNSCC. Additionally, we seek to investigate the function and underlying molecular mechanisms of PDLIM3 involved in HNSCC progression in vitro. Our findings reveal that PDLIM3, which is significantly downregulated in HNSCC, functions as a potential tumor suppressor by inhibiting migration, invasion, and epithelial–mesenchymal transition (EMT). Additionally, it may serve as a prognostic biomarker for HNSCC.

## 2. Results

### 2.1. PDLIM3 Is Downregulated in HNSCC and Correlates with Poor Patient Prognosis

**Figure 1:**
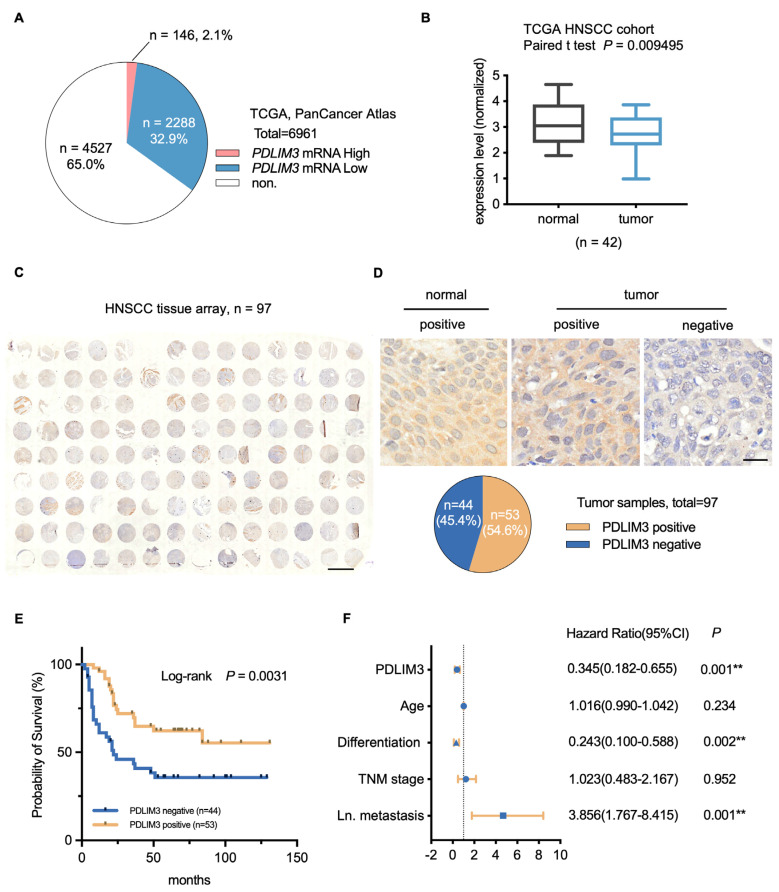
PDZ and LIM domain protein 3 (PDLIM3) are downregulated in head and neck squamous cell carcinoma (HNSCC) and correlate with poor patient prognosis. (**A**) PDLIM3 mRNA expression alteration in pan–cancer based on The Cancer Genome Atlas Program (TCGA) from cBioportal. (**B**) PDLIM3 mRNA expression based on paired samples with tumors and adjacent normal tissues from TCGA HNSCC cohort. (**C**) Anti–PDLIM3 staining of an HNSCC tissue microarray, 20×, scale bar, 2000 μm. (**D**) Top panel: PDLIM3 is positively expressed in normal tissues, and the tumor tissues were categorized into two groups based on staining intensity: negative or positive. Bottom panel: The percentage of PDLIM3 positive or negative in HNSCC samples. Scale bar, 20 μm. (**E**) The Kaplan–Meier plot shows a significantly longer overall survival time in patients with PDLIM3–positive expression compared to those with PDLIM3–negative expression (*p* = 0.0031, Log–rank test). (**F**) Multivariate Cox regression analysis for tissue microarray. Ln. = lymph node. ** *p* < 0.01.

The expression of PDLIM3 was analyzed using data from The Cancer Genome Atlas Program (TCGA) cohort. Dysregulation of PDLIM3 in pan–cancer is primarily characterized by altered mRNA levels, with limited genetic alterations (Figure S1A). Notably, mRNA levels are significantly downregulated in pan–cancer (32.9%, Figure 1A), indicating that PDLIM3 may play a crucial role in tumorigenesis and development. Then, the expression of PDLIM3 in paired samples of HNSCC and normal tissue was compared, and PDLIM3 expression was significantly downregulated in the tumor (Figure 1B).

To investigate the prognostic value of PDLIM3 in HNSCC, 97 cases were evaluated by IHC (Figure 1C). The results showed that PDLIM3 expression was predominantly localized in the cytoplasm. We evaluated the expression of PDLIM3 in 4 normal tissues and 97 HNSCC. Notably, about 45% of the tumor samples were PDLIM3–negative, while PDLIM3 expression was detected in all normal samples (Figure 1D). For survival analysis, the tissues were categorized into two groups based on staining intensity: negative or positive (Figure 1D). There was no significant difference in the expression level of PDLIM3 in the subgroups of age, TNM stage, and tumor stage (Figure S1B and Table 1). The Kaplan–Meier curve (Figure 1E) revealed that patients with high PDLIM3 expression had higher survival rates and longer median survival times compared to those with low expression (Log–rank *p* = 0.0031). Then, Cox analyses were performed to evaluate the independent prognostic value of PDLIM3. The univariate Cox analysis revealed that age (HR = 1.027, *p* = 0.033), differentiation (HR = 0.378, *p* = 0.019), TNM stage (HR = 2.377, *p* = 0.006), lymph node metastasis (HR = 3.122, *p* < 0.001), and PDLIM3 expression (HR = 0.419, *p* = 0.005) may be prognostic factors (Table 2). Furthermore, the multivariate Cox analysis indicated that PDLIM3 (HR = 0.345, *p* = 0.001) may serve as an independent protective factor (Figure 1F). Taken together, our results indicate that PDLIM3 is widely downregulated in pan–cancer, and the downregulation of PDLIM3 in HNSCC correlated with poor patient prognosis. PDLIM3 has the potential to serve as a prognostic factor for HNSCC.

### 2.2. PDLIM3 Inhibits the Migration and Invasion of HNSCC

**Figure 2:**
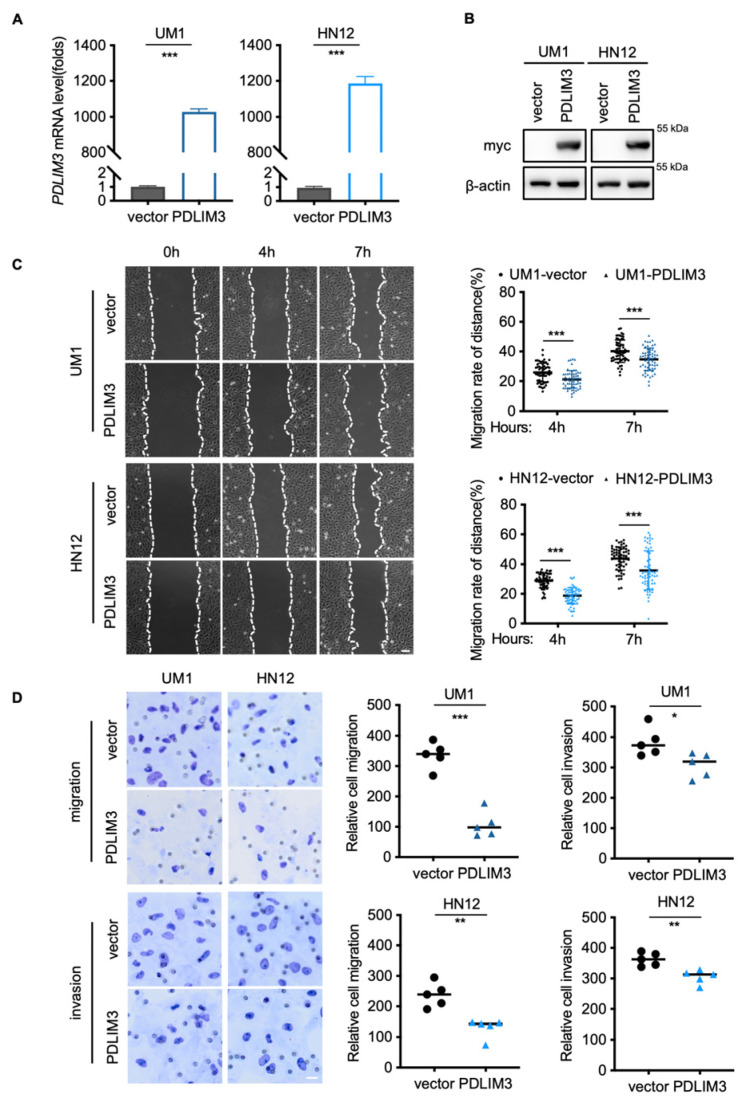
PDLIM3 reduces the migration and invasion of HNSCC in vitro. (**A**) Quantitative real–time PCR (qPCR) confirmed that the UM1 and HN12 cell lines with PDLIM3 overexpression were established using vectors as control. (**B**) Western blotting confirmed the UM1 and HN12 cell lines with myc–tagged PDLIM3 stably expressed are established, using vectors as control. (**C**) Wound healing assay showing cell migration of UM1 and HN12 after 4 h, 7 h, scale bar, 100 μm. (**D**) Transwell assay showing cell migration and invasion of UM1 and HN12 after 24 h, scale bar, 20 μm. * *p* < 0.05, ** *p* < 0.01, and *** *p* < 0.001; ns, not significant.

PDLIM3 expression was assessed in four HNSCC cell lines. The UM1 and HN12 cell lines, which exhibit low expression levels of PDLIM3 (Figure S2A,B), were transduced with lentivirus to stably express myc–tagged PDLIM3. To investigate the function of PDLIM3 in HNSCC, we first monitored cell numbers in real–time over three proliferation cycles and found that PDLIM3 had little effect on the proliferative ability of HNSCC cell lines (Figure S2C). Similarly, no significant effect of PDLIM3 expression on cell growth was observed in the EdU assay (Figure S2D). We found that cell lines with low expression of PDLIM3 exhibit a strong capacity for migration (Figure S2E). Then, we explored whether PDLIM3 contributes to cellular motility in HNSCC cell lines. Wound healing and transwell assays showed that PDLIM3 inhibits both cell migration and invasion in vitro (Figure 2C,D). Together, PDLIM3 reduces the migratory and invasive capabilities of HNSCC in vitro rather than affecting cell proliferation.

### 2.3. PDLIM3 Partially Impeding the Process of EMT in HNSCC

**Figure 3:**
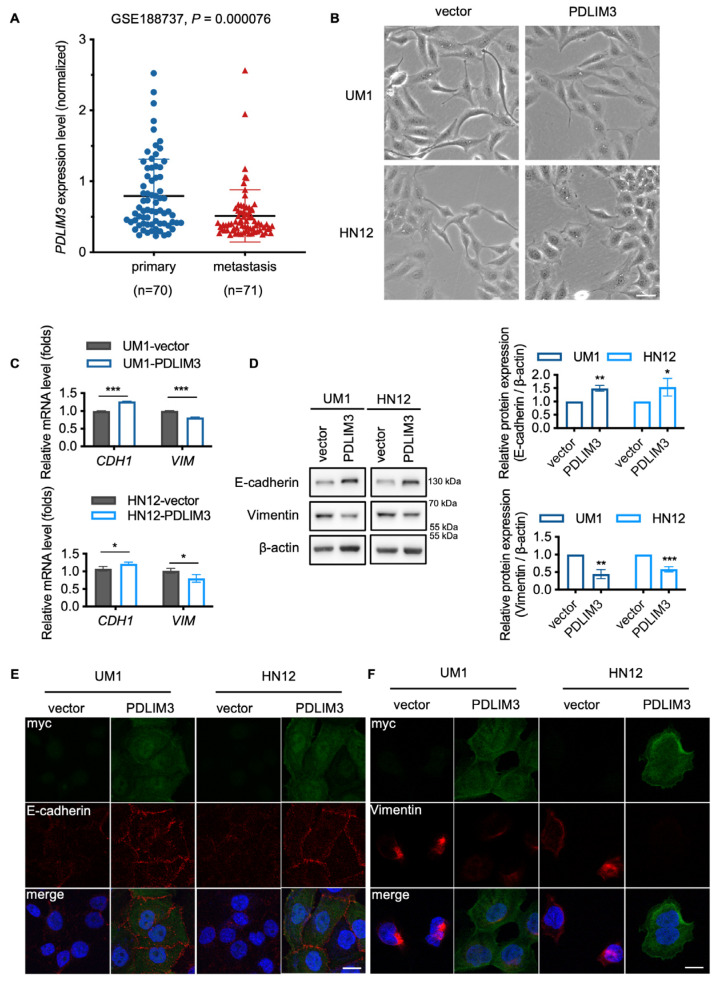
PDLIM3 partially inhibits the process of epithelial–mesenchymal transition (EMT) in HNSCC. (**A**) PDLIM3 expression in HNSCC patients with or without metastasis from GSE188737. (**B**) Representative morphology of vector and PDLIM3 expression cells after being seeded at the same density for the same duration, scale bar, 100 μm. (**C**) Western blotting reveals the influence of PDLIM3 on the expression of EMT–related molecules in HNSCC cells. (**D**) qPCR reveals the influence of PDLIM3 on the expression of EMT–related genes in HNSCC cells. (**E**) Cells were stained by immunofluorescence to detect the expression of the epithelial marker E–cadherin localization of cell membranes. Scale bar, 20 μm. (**F**) Cells were stained by immunofluorescence to detect the expression of the mesenchymal marker Vimentin localization of cytoplasm. Scale bar, 20 μm. * *p* < 0.05, ** *p* < 0.01 and *** *p* < 0.001.

An analysis of a public dataset containing information on tumor metastasis in HNSCC patients (GSE188737) revealed that PDLIM3 was significantly downregulated in patients with metastasis (Figure 3A). This finding suggests a potential negative correlation between PDLIM3 expression and tumor metastatic ability, consistent with in vitro experiments (Figure S2E). The EMT programme is one of the crucial contributors to tumor invasiveness and metastatic ability [21,22,23]. We sought to determine whether PDLIM3 expression could impact the process of EMT in HNSCC cells.

Compared to the vectors, cells expressing PDLIM3 exhibited an epithelial–like phenotype. Specifically, there were fewer edge protrusions and pseudopodia, a reduction in fibroblast–like morphology, and tighter adhesion and aggregation (Figure 3B). Western blotting and qPCR were performed to examine the change of EMT–related molecules. Consistent with the observed morphological changes, the epithelial marker E–cadherin (*CDH1*) was upregulated, while the mesenchymal marker Vimentin (*VIM*) was downregulated (Figure 3C,D). Immunofluorescence assays directly showed the impact of PDLIM3 on EMT. With myc–tagged PDLIM3 positively expressed in the cytoplasm, cells accompanied by E–cadherin expression were increased, and Vimentin was downregulated (Figure 3E,F). Together, these results indicated that PDLIM3 partially inhibited the process of EMT in HNSCC.

### 2.4. The Potential Role of Yes–Associated Protein (YAP) in PDLIM3–Mediated Suppression of EMT in HNSCC

To explore the potential molecular mechanisms by which PDLIM3 exerts its biological functions in HNSCC, the TCGA samples were classified into PDLIM3 high group and PDLIM3 low group based on the median value of PDLIM3 expression. The volcano plot illustrates the differential genes between the two groups (Figure S3A). Gene ontology (GO) terms and Kyoto Encyclopedia of Genes and Genomes (KEGG) pathway enrichment analysis revealed that differential genes were mainly enriched in extracellular matrix (ECM)–receptor interaction, extracellular matrix, cell adhesion (Figure S3B,C). This suggests that PDLIM3–related genes are closely associated with the cytoskeleton and the process of EMT in HNSCC.

**Figure 4:**
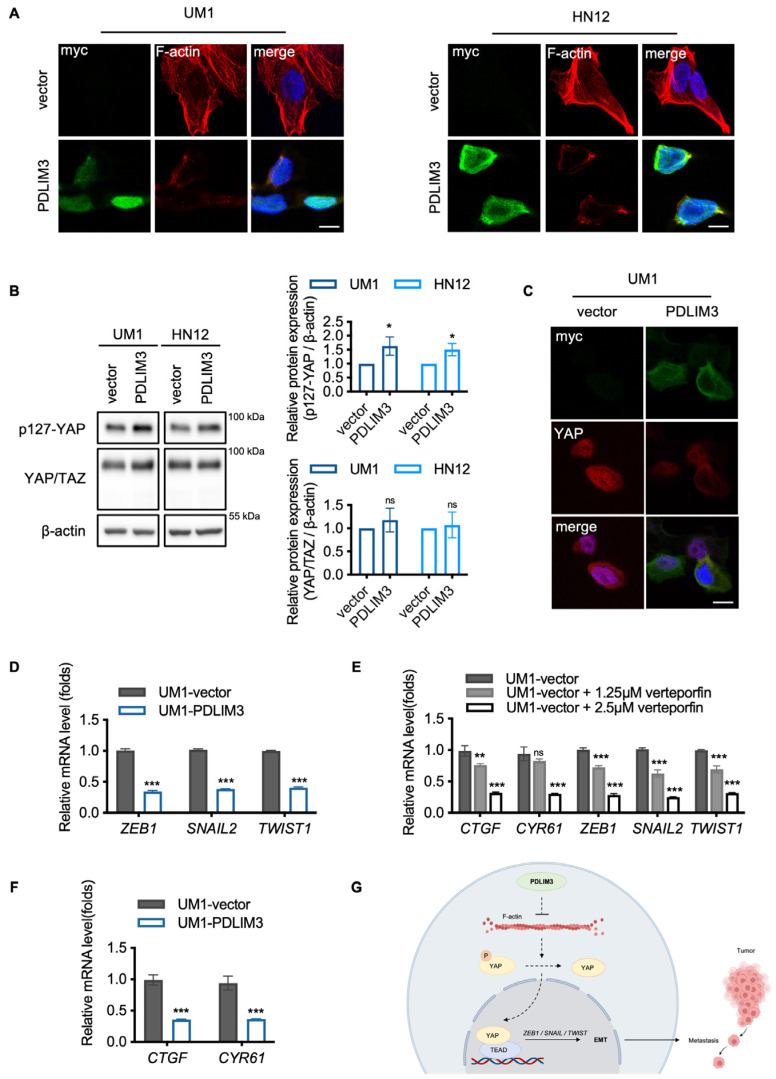
The potential role of Yes–associated protein (YAP) in PDLIM3–mediated suppression of EMT in HNSCC. (**A**) Cells were stained by immunofluorescence to detect the expression of the F–actin localization of cytoplasm. Scale bar, 20 μm. (**B**) Western blotting indicates that the phosphorylation level of YAP at serine 127 is increased by PDLIM3. (**C**) Cells were stained by immunofluorescence to detect the expression of the YAP, and the nuclear positivity or nuclear translocation was inhibited by PDLIM3. 40×, scale bar, 20 μm. (**D**) qPCR reveals the effect of PDLIM3 on YAP target genes. (**E**) qPCR shows the mRNA levels of YAP and the EMT transcription factors (EMT–TFs) in vector cells treated with the YAP inhibitor verteporfin (VP) at the indicated concentration for 24 h, using UM1–vector as control. (**F**) qPCR reveals the effect of PDLIM3 on the EMT–TFs. (**G**) Mechanism schematic illustrating. Cartoon depicting a potential regulatory axis of YAP–EMT regulated by PDLIM3 in HNSCC. In detail, PDLIM3 inhibits the EMT–TFs by suppressing YAP, thereby inhibiting tumor migration and invasion. See text for details. * *p* < 0.05, ** *p* < 0.01, and *** *p* < 0.001; ns, not significant.

The process of EMT typically involves the rearrangement of F–actin conformations [24,25], which enhances the migratory and invasive capabilities of cells. However, F–actin was inhibited in cells expressing PDLIM3 (Figure 4A). Previous studies have shown that F–actin can activate YAP independently of the Hippo signaling [26,27]. YAP is uniquely positioned as a key link between cytoskeletal changes and transcriptional programs. It can directly regulate core EMT transcription factors (EMT–TFs), such as *ZEB1*, *SNAIL*, and *TWIST*, and promote tumor metastasis by activating EMT [24,28,29,30,31,32,33]. This prompted us to investigate whether YAP is involved in the regulation of EMT by PDLIM3. Western blotting and immunofluorescence were performed to investigate the potential regulatory relationship between PDLIM3 and YAP. Our findings suggest that the phosphorylation of YAP at ser127 was increased (Figure 4B), and nuclear translocation of YAP was inhibited by PDLIM3 overexpression (Figure 4C). Taken together, PDLIM3 inhibits YAP transcriptional activity.

The downregulation of YAP targeting genes *CTGF* and *CYR61* further validated the inhibitory effects of PDLIM3 on YAP (Figure 4D). Furthermore, to explore the role of YAP in the regulation of EMT in HNSCC, UM1 was treated with verteporfin (VP), an inhibitor of the TEAD–YAP interactions [34]. *CTGF*, *CYR61*, and the EMT–TFs, including *ZEB1*, *SNAIL*, and *TWIST*, which are directly regulated by YAP [24,28], were downregulated in correlation with the degree of YAP inhibition (Figure 4E). These results validate that the EMT transcriptional program was suppressed by inhibition of YAP in HNSCC. Moreover, the EMT–TFs were downregulated by PDLIM3 overexpression (Figure 4F). Collectively, our results reveal that PDLIM3 inhibits YAP transcriptional activity, suggesting YAP may be involved in the PDLIM3–mediated suppression of EMT in HNSCC.

## 3. Discussion

HNSCC is prone to recurrence or metastasis, which are major contributors to poor prognosis and postoperative treatment failure [7,8]. Investigating the aberrant molecular events in tumors enhances our understanding of cancer progression and metastasis, thereby providing a theoretical foundation for developing targeted therapeutic strategies and improving patient prognosis [11,35].

Our study identifies that PDLIM3 is downregulated in HNSCC and underscores its significant role as a suppressor in EMT, migration, and invasion, suggesting that PDLIM3 has considerable potential in regulating the metastatic capabilities of HNSCC. Numerous studies have reported abnormal expression of PDLIM3 across various cancers, including its upregulation in gastric cancer [19], colon cancer [36], and the SHH subgroup of medulloblastoma [18], while significant downregulation has been found in prostate cancer [20]. In the SHH subgroup of medulloblastoma, PDLIM3 expression is significantly higher than in normal brain tissue, and its loss impairs cilia formation, inhibiting tumor cell proliferation and growth [18]. Our study identifies PDLIM3 as a novel suppressor of EMT and metastatic ability in HNSCC, distinct from its oncogenic roles in gastric and medulloblastoma cancers. On the one hand, this dual role suggests the tissue–specific regulation of PDLIM3, with different regulatory functions in proliferation and migration depending on the tissue of origin [12]. On the other hand, epigenetic mechanisms [37,38,39] or specific microRNA [40] regulation may contribute to the distinct expression patterns of PDLIM3 across different cancers. Therefore, further studies are worthwhile to elucidate its upstream regulators and interaction networks in different tissues and cancers. Moreover, the roles and regulatory mechanisms of PDLIM3 in HNSCC have not been fully explored. Our research demonstrates that PDLIM3 functions as a suppressor in HNSCC accompanied by downregulating EMT–related molecules and inhibiting migration and invasion in vitro. Notably, a previous study compared differences in PDLIM3 expression across T–stages and cervical lymph node metastasis [41]. They found elevated PDLIM3 expression in tongue cancer with early regional lymph node metastasis. In our study, the correlation between PDLIM3 and T–stage was not statistically significant (Table 1). The high heterogeneity of HNSCC may be a potential reason for this discrepancy. Additionally, variations in the type of cancers, sample sources, sampling methods, and limited sample sizes may introduce bias into the results. Therefore, functional experiments are needed to verify these conclusions. Undeniably, we both propose that PDLIM3 is associated with the metastasis of HNSCC. Therefore, further in vivo experiments are necessary to confirm this in the future.

Beyond PDLIM3, other PDLIM family proteins have similar functions and participate in tumor progression. Low PDLIM1 expression correlates with metastasis and poor prognosis in liver cancer, where it limits F–actin formation and reverses EMT, suppressing hepatocellular carcinoma metastasis [42]. Overexpression of PDLIM2 in THP–1 cells enhanced cell adhesion [43]. Similarly, PDLIM4 is associated with poor prognosis in ovarian cancer by inhibiting tumor invasion through the suppression of STAT3 signaling [38]. Thus, PDLIM family proteins hold significant promise as therapeutic targets in cancer treatment. Studying the roles of PDLIM family proteins in specific tumors is crucial for understanding tumor development and metastasis, as well as for developing corresponding therapeutic strategies.

Additionally, our study documents show that YAP may play a key role in the inhibition of EMT by PDLIM3. As a transcriptional co–activator, YAP is a pivotal regulator of gene networks [31]. In approximately 80% of HNSCC cases, YAP is abnormally activated [44,45], preventing normal tumor cell differentiation and being associated with increased invasiveness and poorer prognosis [31]. YAP activation enhances EMT, allowing cancer cells to acquire migratory and invasive characteristics that facilitate metastasis [31]. In our study, we observed that PDLIM3 can depolymerize F–actin, an important upstream regulator of YAP, prompting us to hypothesize a regulatory relationship between PDLIM3 and YAP. Our results confirmed that PDLIM3 inhibits the transcriptional activity of YAP in vitro. Moreover, both PDLIM3 and verteporfin (VP) suppressed the EMT transcriptional program, indicating the inhibitory roles of PDLIM3 and YAP in EMT in HNSCC. Previous research has also reported that PDLIM1 can inhibit YAP, thereby suppressing hepatocellular carcinoma metastasis [42]. Based on existing studies and experimental evidence, it is reasonable to propose that YAP is the key mediator through which PDLIM3 inhibits EMT and metastatic ability in HNSCC.

Collectively, our findings reveal that PDLIM3 correlates with poor prognosis, suggesting that it may serve as a valuable prognostic biomarker for risk stratification in HNSCC. Furthermore, our findings suggest that PDLIM3 acts as a tumor suppressor in HNSCC by inhibiting migration and invasion. Given that PDLIM3 lacks intrinsic kinase activity, exploring targetable activators upstream of PDLIM3 to modulate its function may provide new strategies for precision therapy in cases of PDLIM3 dysregulation. Additionally, both the downstream pathways of YAP and EMT are challenging to target directly in precision therapy [23,46,47]. Therefore, considering the role of PDLIM3 in cytoskeletal remodeling, we propose to further investigate its interactions with cytoskeletal–related druggable targets, such as focal adhesion kinase (FAK) [48] and Rho–associated protein kinase (ROCK) [49,50], which may provide valuable insights for clinical applications.

## 4. Materials and Methods

### 4.1. Tissue Microarray Analysis

Tissue samples were collected at West China Hospital of Stomatology for a cohort study with regular follow–up. The cohort study was conducted in accordance with The Code of Ethics of the World Medical Association (Declaration of Helsinki) for experiments involving humans, with the approval of the Scientific and Ethics Committee of West China Hospital of Stomatology, Sichuan University, China. Informed consent was obtained from all patients or their relatives for the collection and use of tissues in the study. During the follow–up period, researchers comprehensively recorded the clinical information and medical history of patients, including age, gender, smoking status, alcohol consumption, tumor differentiation, TNM stages, and tumor metastasis status, along with the assurance of protecting the privacy rights of human subjects all the time. Survival time was recorded from the day of surgery until cancer–related death or the end of the follow–up period (the median follow–up time was 69 months, 95%CI 62.132–75.868 months). In this study, tissue samples were collected from four healthy individuals and 122 HNSCC patients. After immunohistochemistry staining, 97 tumor samples with identifiable tumor cells were subjected to analysis.

### 4.2. Cell Line and Cell Culture

HNSCC Cell lines (CAL27, SCC4, UM1, HN12) and HEK293T were obtained from the National Key Laboratory of Oral Diseases, Sichuan University. The provenances of all cell lines have been previously documented [44,51,52]. All cell lines were cultured in Dulbecco’s Modified Eagle Medium (DMEM; Hyclone, SH30243.01, Logan, UT, USA), supplemented with 10% fetal bovine serum (FBS; PAN, ST30–3302, Aidenbach, Germany) and 1% penicillin–streptomycin (Biosharp, BL505A, Hefei, China), at 37 °C with 5% CO_2_. The experiments started after two passages after cell recovery. Cell passaging was performed when the cell confluence was about 70–80, and the number of cell passages did not exceed 15.

### 4.3. Plasmid Construction, Lentivirus Production, and Stable Cell Line Construction

Method descriptions have been previously described [51]. The plasmid synthesized in this study was generated using PrimeSTAR Max DNA Polymerase (Takara, R045A, Kyoto, Japan) and HiFi DNA Assembly Master Mix (NEB, E2621, Ipswich, MA, USA) via Gibson Assembly. Gene comprising 3× myc–PDLIM3 were subcloned and integrated into the pCDH–puro vector (SBI, CD510B–1, Palo Alto, CA, USA). Transfection was performed using Lipofectamine 2000 (Thermo Fisher Scientific, 11668019, Waltham, MA, USA). For lentivirus production, HEK293T cells were co–transfected with pCDH–based plasmids alongside PsPAX2 and pMD2.g. After 48 h, the medium containing the lentiviral particles was filtered and used for viral transduction.

Due to the low expression levels of PDLIM3, we selected UM1 and HN12 cells for gain–of–function experiments. For the construction of stable cell lines, cells were inoculated in 6–well plates, and lentiviral vector transduction was performed using Polybrene (10 μg/mL, Santa Cruz, sc–134220, Dallas, TX, USA) and lentivirus for 24 h, with a multiplicity of infection (MOI) of 3. Cells infected with an empty lentiviral vector served as the control. Following this, puromycin (1.5 μg/mL, Selleck, S7417, Houston, TX, USA) was used for the selection of stable transgenic cells. Subsequently, the transduction efficiency was verified using qPCR and Western blot assays.

### 4.4. Quantitative Real–Time PCR (qPCR) Analysis

Total RNA from the cell lines was isolated using the RNeasy Mini Kit (FORE GENE, RE 03111, Chengdu, China). RNA concentrations were determined using NanoDrop, and cDNA was synthesized using the PrimeScript™ RT reagent Kit (Perfect Real Time) (Takara, RR037A, Japan) according to the manufacturer’s instructions. The relative expression of RNA was evaluated by qPCR analysis using the SYBR Green method, performed in triplicate. *GAPDH* was used as an internal control for mRNA quantification. Relative RNA expression levels were calculated using the comparative Ct method. The primer sequences are shown in Table 3.

### 4.5. Western Blotting

Western blotting was performed as previously described [53]. In brief, cells were lysed using 1× lysis buffer. The lysates were sonicated, boiled, and loaded for electrophoresis, then transferred onto the PVDF membrane (Millipore, ISEQ00010, St. Louis, MO, USA). The membranes were blocked with 5% nonfat milk at room temperature. Primary antibodies were incubated overnight at 4 °C. The following day, membranes were incubated with secondary antibodies at room temperature, and protein bands were visualized using a chemiluminescence solution (Millipore, WBKLS0500).

The primary antibodies used were as follows: anti–myc (Cell Signaling Technology, 2276, Danvers, MA, USA) 1:4000, anti–PDLIM3 (Proteintech, 13199–2–AP) 1:4000, anti–E–cadherin (Cell Signaling Technology, 14472) 1:3000, anti–Vimentin (Cell Signaling Technology, 5741) 1:3000, anti–p–YAP(Ser127) (Cell Signaling Technology, 13008) 1:2000, anti–YAP (Cell Signaling Technology, 93622) 1:4000, anti–beta–actin (Santa Cruz, sc–69879, Dallas, TX, USA) 1:4000. HRP–conjugated goat anti–rabbit (ZSGB–BIO, ZB2301, Beijing, China) and anti–mouse IgGs (ZSGB–BIO, ZB2305) were used at a dilution of 1:10,000.

### 4.6. In Vitro Cell Proliferation Assay

Cells were seeded in 24–well plates at approximately 5% confluency per well to conduct cell proliferation experiments, with three replicate wells per group. Cells were cultured in a medium containing 1% FBS at 37 °C with 5% CO_2_. Proliferation was monitored over 72 h using the Cytation5 imaging system (Agilent, Santa Clara, CA, USA), with images captured and analyzed every 4 h using a 4× objective lens.

### 4.7. 5–Ethynyl–2′–Deoxyuridine (EdU) Assay

EdU assay was performed using the BeyoClick™ EdU Cell Proliferation Kit with Alexa Fluor 594 (Beyotime, C0078S, Shanghai, China). In brief, cells were seeded in a 24–well plate 24 h before the assay, with three replicate wells per cell type. After washing with PBS, cells were incubated with EdU solution for 2 h and then stained with Hoechst solution for nuclear staining. Samples were observed and photographed using an inverted microscope (Olympus, Tokyo, Japan).

### 4.8. Wound Healing Assay

A wound healing assay was performed as previously described [52]. Cells were seeded in 6–well plates at 80% confluency and grown overnight until reaching full confluence. Subsequently, they were pre–treated with 2 μM mitomycin C (Selleck, S8146, USA) for 1 h to inhibit cell proliferation. A 200 μL sterile pipette tip was used to create a linear scratch in the middle of each well. Cells were then washed with PBS, and serum–free medium was added. Images were captured at the appropriate time. The migration distance was measured using ImageJ, and the migration rate was calculated as (scratch width at 0 h–scratch width at the specific time point)/scratch width at 0 h.

### 4.9. Transwell Assay

Transwell assay was performed as previously described [51]. In detail, for the migration assay, 8 μm transwell (BD Bioscience, 353097, Milpitas, CA, USA) inserts were placed in 24–well plates. The upper chamber was filled with 200 μL of serum–free cell suspension at a concentration of 4 × 105 cells/mL, and the lower chamber contained 700 μL of complete medium. After 24 h of incubation, cells were fixed with 4% paraformaldehyde and stained with crystal violet. Membranes were removed and mounted with neutral gum, and the results were scanned using VS200 (Olympus, Japan) and analyzed with ImageJ (version 2.1.0).

For the invasion assay, Matrigel (Corning, 356234, Corning, NY, USA) was diluted with a serum–free medium at a 1:8, and 37 μL of the mixture was added to each transwell chamber, then incubated at 37 °C for 1 h, with the subsequent steps being identical to those of the migration assay.

### 4.10. Immunofluorescence

Cells were seeded on chamber slides, fixed with 4% paraformaldehyde containing 2% sucrose, permeabilized with 0.25% Triton X–100 (Sigma, T8787, St. Louis, MO, USA), blocked with 3% bovine serum albumin (BSA), and incubated overnight at 4 °C with primary antibodies diluted in 4 mg/mL BSA. The secondary antibodies were incubated for 1 h at room temperature in the dark, followed by incubation with phalloidin (1:100; Beyotime, C2203S, China) for 30 min at room temperature in the dark. Slides were mounted with DAPI, and images were captured using a confocal Stellaris8 (Leica, Wetzlar, Germany).

### 4.11. Immunohistochemistry

Immunohistochemistry assays were conducted to detect PDLIM3 using a primary antibody, following antigen retrieval with citrate buffer (0.01 M, pH 6.0). Protocol was performed as previously described [44]. To determine the level of PDLM3 expression in the HNSCC clinical cohort, staining intensity was evaluated (0 for negative staining, 1 for positive staining). This assessment was performed by two proficient pathologists who were unaware of the clinical and pathological details (Kappa = 0.747, *p* < 0.0001). If the conclusions are inconsistent, the final result will be determined through a joint review and discussion.

### 4.12. Third–Party mRNA Sequencing Analysis

Bulk RNA–seq data for the TCGA HNSCC cohort (paired tumor and normal samples, *n* = 42) were obtained from cBioPortal (https://cbioportal–datahub.s3.amazonaws.com/hnsc_tcga_pan_can_atlas_2018.tar.gz, accessed on 6 September 2024). PDLIM3 expression levels were log10 normalized, and a paired Student t–test was used to compare expression differences between tumor and normal tissues.

The TCGA HNSCC samples were classified into PDLIM3 high group and PDLIM3 low group based on the median value of PDLIM3 expression. An adjusted *p*–value of less than 0.05 was established as the criterion for differential gene screening, while a fold difference of greater than 2 in the PDLIM3 high–expression group compared to the low–expression group was used as the threshold for significant upregulation and a fold difference of less than 0.5 was used as the threshold for significant downregulation. Besides, GO and KEGG enrichment analyses of differential genes were performed.

A scRNA–seq dataset and corresponding annotation data from primary (*n* = 70) and metastatic HNSCC patients (*n* = 71) were acquired from a previous study [54]. PDLIM3 expression levels of primary and metastatic tumor cells were normalized using the NormalizeData function in the Seurat package (https://www.nature.com/articles/s41587–023–01767–y, accessed on 12 August 2024) and compared using a *t*–test.

### 4.13. Statistical Analysis

Statistical analyses were performed using SPSS Statistics 26 and GraphPad Prism 10. Continuous variables were presented as mean ± standard deviation (SD). Each experiment was performed a minimum of three times. The normality test was conducted using GraphPad Prism 10 and evaluated using the Shapiro–Wilk test. Parametric or nonparametric tests were used according to the normality of the data. Two–tailed Student’s t–tests were used for comparisons between two groups, and one–way analysis of variance (ANOVA) with Bonferroni correction was used for comparisons among multiple groups. The Kaplan–Meier curve was performed using the survival data from the tissue array, and differences among groups were analyzed by the Log–rank test. Univariable and multivariable Cox regression analyses were conducted to calculate the hazard ratio (HR) and 95% confidence interval (CI) to identify independent prognostic clinical and pathological factors using IBM SPSS Statistics 26. Statistical significance was defined as *p* < 0.05. * *p* < 0.05; ** *p* < 0.01; and *** *p* < 0.001; ns, not significant.

## Figures and Tables

**Table 1:** Correlation of PDLIM3 in the study population with clinicopathological factors, including patients and tumor characteristics (*n* = 97).

Variable	Patients	PDLIM3		χ²	*p*
	*n* (%)	Positive	Negative		
Age					
≤60 years	43 (44)	20	23	0.04127	0.8390
>60 years	54 (56)	24	30		
Gender					
Male	63 (65)	30	33	0.3698	0.5431
Female	34 (35)	14	20		
History of smoking					
No	59 (61)	32	27	0.0098	0.9211
Yes	38 (39)	21	17		
History of alcohol					
No	67 (69)	36	31	0.07204	0.7884
Yes	30 (31)	17	13		
Differentiation					
High	68 (70)	30	38	2.595	0.2732
Moderate	24 (25)	10	14		
Low	5 (5)	4	1		
TNM stage					
I/II	39 (40)	14	25	2.357	0.1247
III/IV	58 (60)	30	28		
Tumor stage					
T1/T2	61 (63)	26	35	0.4971	0.4808
T3/T4	36 (37)	18	18		
Lymph node metastasis					
No	53 (55)	20	33	2.741	0.0978
Yes	44 (45)	24	20		
Radiotherapy					
No	82 (85)	43	39	1.036	0.3088
Yes	15 (15)	10	5		
Pre–Chemotherapy					
No	66 (68)	43	23	9.208	0.0024
Yes	31 (32)	10	21		
Post–Chemotherapy					
No	50 (52)	25	25	0.8961	0.3438
Yes	47 (48)	28	19		

**Table 2:** Cox regression analysis of prognostic factors in tissue array samples.

	Univariate Cox Regression Analysis			Multivariate Cox Regression Analysis		
	HR	95% CI	*p*–Value	HR	95% CI	*p*–Value
PDLIM3	0.419	0.229–0.764	0.005	0.345	0.182–0.655	0.001
Age	1.027	1.002–1.053	0.033	1.016	0.990–1.042	0.234
Gender	0.640	0.329–1.244	0.188			
Differentiation	0.378	0.168–0.850	0.019	0.243	0.100–0.588	0.002
TNM stage	2.377	1.279–4.417	0.006	1.023	0.483–2.167	0.952
Tumor stage	1.928	0.922–4.034	0.081			
Lymph node metastasis	3.122	1.667–5.849	<0.001	3.856	1.767–8.415	0.001

**Table 3:** Primer Sequence of RT–qPCR analysis.

**Gene**	**Forward (5′–3′)**	**Reverse (5′–3′)**
*GAPDH*	GAGTCAACGGATTTGGTCGT	TTGATTTTGGAGGGATCTCG
*PDLIM3*	AACTCGCCAATTGGGCTCTA	CTGGAGCACTCTGAAGGAGC
*CDH1*	CGAGAGCTACACGTTCACGG	GGGTGTCGAGGGAAAAATAGG
*VIM*	TGCCGTTGAAGCTGCTAACTA	CCAGAGGGAGTGAATCCAGATTA
*CTGF*	GTTTGGCCCAGACCCAACTA	GGCTCTGCTTCTCTAGCCTG
*CYR61*	CAGGACTGTGAAGATGCGGT	GCCTGTAGAAGGGAAACGCT
*ZEB1*	GATGATGAATGCGAGTCAGATGC	ACAGCAGTGTCTTGTTGTTGT
*SNAIL2*	CGAACTGGACACACATACAGTG	CTGAGGATCTCTGGTTGTGGT
*TWIST1*	GTCCGCAGTCTTACGAGGAG	GCTTGAGGGTCTGAATCTTGCT

## Data Availability

The public datasets are available online. See Materials and Methods for details. The data that support the findings of this study are available from the corresponding author upon reasonable request.

## Supplementary Materials

The following supporting information can be downloaded at https://www.mdpi.com/article/10.3390/ijms26073147/s1.
