# Supplementary material for: PDLIM3 Regulates Migration and Invasion of Head and Neck Squamous Cell Carcinoma via YAP–Mediated Epithelial–Mesenchymal Transition
Source: Int J Mol Sci. 2025 Mar 28;26(7):3147. doi: 10.3390/ijms26073147 (PMC11988593; doi:10.3390/ijms26073147)
Supplement: Supplementary file 1 [file ijms-26-03147-s001.zip › ijms-3504315-supplementary.pdf]

International Journal of *Molecular Sciences*

MDPI

Supplementary Information

PDLIM3 Regulates Migration and Invasion of Head and Neck Squamous Cell Carcinoma via YAP-Mediated Epithelial-Mesenchymal Transition

Fan Yang [1], Ying Zhou [1], You Zhang [1], Weideng Wei [1], Fei Huang [1], Dan Yang [1], Yixin Zhang [1], Ruiyang Zhang [1], Xiaoqiang Xia [1], Qianming Chen [2], Yuchen Jiang [1,*] and Xiaodong Feng [1,*]

1 State Key Laboratory of Oral Diseases & National Center for Stomatology & National Clinical Research Center for Oral Diseases & Frontier Innovation Center for Dental Medicine Plus & Research Unit of Oral Carcinogenesis and Management & Chinese Academy of Medical Sciences, West China Hospital of Stomatology, Sichuan University, Chengdu 610041, Sichuan, China; fanyang2022@stu.scu.edu.cn (F.Y.); 2020324030006@alu.scu.edu.cn (Y.Z.); u20230320@outlook.com (Y.Z.); weiweideng@stu.scu.edu.cn (W.W.); antigenjerry@163.com (F.H.); ydan1@stu.scu.edu.cn (D.Y.); 2020224035084@stu.scu.edu.cn (Y.Z.); ruiyang0910@163.com (R.Z.); XXQ_312@163.com (X.X.); qmchen@scu.edu.cn (Q.C.)

* Correspondence: xiaodongfeng@scu.edu.cn (X.F.); jiangyuchen16@scu.edu.cn (Y.J.)

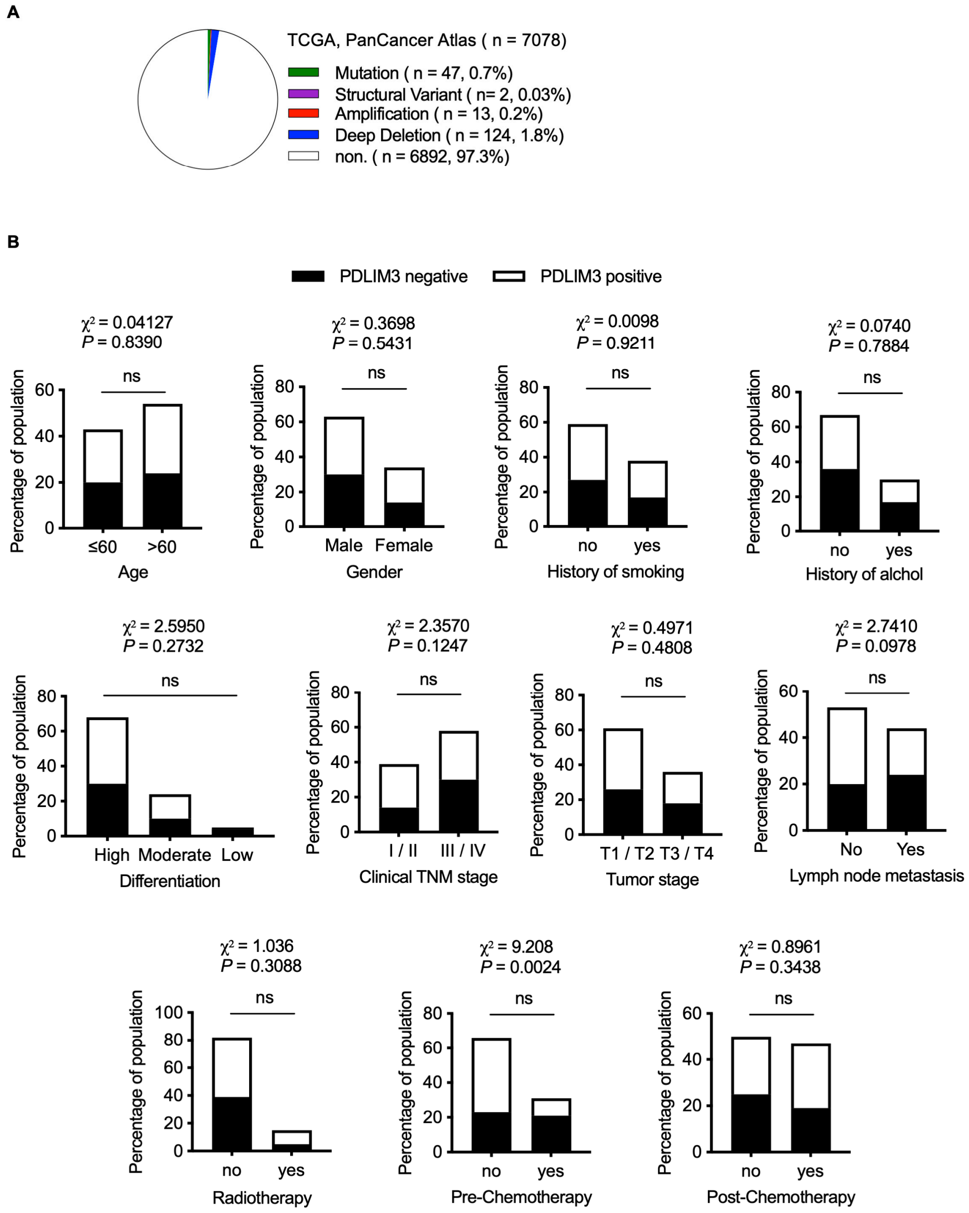

Figure S1. (A) *PDLIM3* genetic alteration in pan-cancer based on TCGA from cBioportal. (B) The difference of PDLIM3 expression among patient demographic characteristics and disease progression in tissue array.

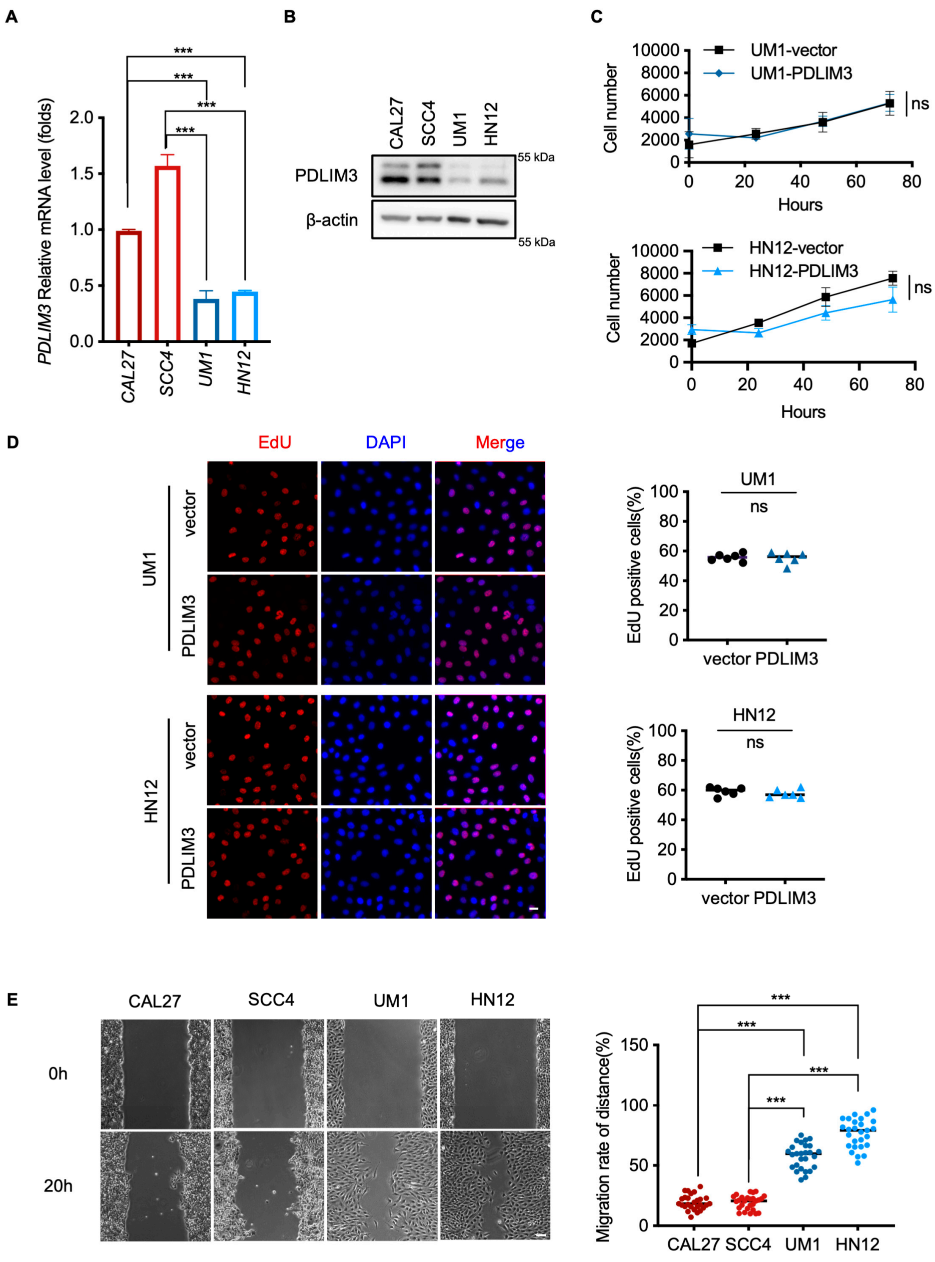

Figure S2. (A) PDLIM3 mRNA levels in HNSCC cell lines. (B) PDLIM3 protein expression in HNSCC cell lines. (C) The cell numbers of UM1 and HN12 are monitored using a viable cell counter last 72 hours. (D) EdU assays shows the difference of cell proliferation between PDLIM3 expression and vector. (E) Wound healing assay showing cell migration of HNSCC cell lines after 20h, scale bar, 100μm.

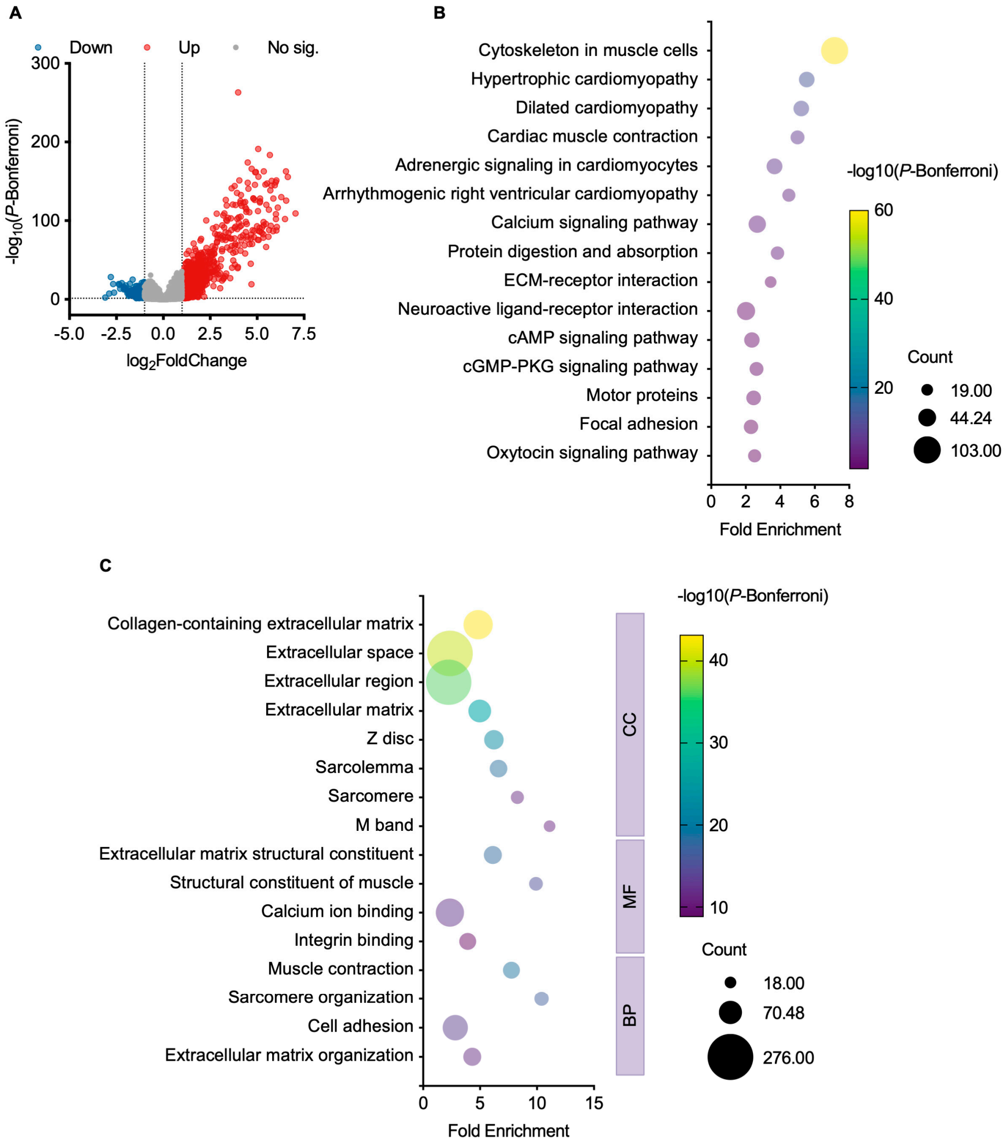

Figure S3. PDLIM3-related genes are closely associated with the cytoskeleton and the process of EMT in HNSCC. (A) The volcano plot shows the differential genes between PDLIM3 high group and low group. (B) Gene ontology (GO) terms and (C) Kyoto Encyclopedia of Genes and Genomes (KEGG) pathways of differential genes between PDLIM3 high group and low group. CC, cellular component; BP, biological process; MF, molecular function.
